# German normative data with naming latencies for 283 action pictures and 600 action verbs

**DOI:** 10.3758/s13428-021-01647-w

**Published:** 2021-08-02

**Authors:** Johannes L. Busch, Femke S. Haeussler, Frank Domahs, Lars Timmermann, Immo Weber, Carina R. Oehrn

**Affiliations:** 1 Department of Neurology, Philipps-University Marburg, Marburg, Germany; 2 Department of Linguistics, University of Erfurt, Erfurt, Germany; 3 Center for Mind, Brain and Behavior (CMBB), Philipps-University Marburg, Marburg, Germany

**Keywords:** Action naming, Picture naming, Verbs, Normative data, Name agreement, Motor content, German

## Abstract

Timed picture naming is a common psycholinguistic paradigm. In this task, participants are asked to label visually depicted objects or actions. Naming performance can be influenced by several picture and verb characteristics which demands fully characterized normative data. In this study, we provide a first German normative data set of picture and verb characteristics associated with a compilation of 283 freely available action pictures and 600 action verbs including naming latencies from 55 participants. We report standard measures for pictures and verbs such as name agreement indices, visual complexity, word frequency, word length, imageability and age of acquisition. In addition, we include less common parameters, such as orthographic Levenshtein distance, transitivity, reflexivity, morphological complexity, and motor content of the pictures and their associated verbs. We use repeated measures correlations in order to investigate associations between picture and word characteristics and linear mixed effects modeling for the prediction of naming latency. Our analyses reveal comparable results to previous studies in other languages, indicating high construct validity. We found that naming latency varied as a function of entropy of responses, word frequency and motor content of pictures and words. In summary, we provide first German normative data for action pictures and their associated verbs and identify variables influencing naming latency.

**Supplementary Information:**

The online version contains supplementary material available at 10.3758/s13428-021-01647-w.

## Introduction

Language processing is an intensively studied subject in the field of human behavior and neuroscience. While a considerate amount of research has been devoted to language comprehension, language production is relatively understudied, partially due to the lack of well-characterized stimulus material (for review see Harley, 2014). A well-established task to study language production is timed picture naming (Cattell, 1886; Oldfield & Wingfield, 1964). During the task, participants view pictorial stimuli and label the depicted scene with one word. Behavioral performance is mainly evaluated as naming latency and—in the case of predetermined reference answers—correctness of responses. Using this task, effects of particular picture characteristics or attributes of the associated words on language production can be studied.

There is a large body of evidence on behavioral and neural correlates of labeling visually presented objects, i.e., producing the associated nouns (Bates et al., 2003; Glaser, 1992; Indefrey, 2011; Perret & Bonin, 2018). However, studies indicate that verb and noun processing are subserved by distinct behavioral and neural processes (Mätzig, Druks, Masterson, & Vigliocco, 2009; Vigliocco, Vinson, Druks, Barber, & Cappa, 2011). In contrast to object naming, the production of verbs by describing visually presented actions (action naming) is relatively understudied. This is partly due to the lack of well-characterized stimulus material. While data sets for object naming are widely available, few studies report psycholinguistic variables and naming latencies for pictorial action stimuli (Cuetos & Alija, 2003; Khwaileh, Mustafawi, Herbert, & Howard, 2018; Schwitter, Boyer, Méot, Bonin, & Laganaro, 2004; Shao, Roelofs, & Meyer, 2014; Székely et al., 2005). However, such resources are important for matching stimulus sets for different experimental conditions or groups. Pictorial stimuli may differ among a range of properties which are known to affect naming performance (Perret & Bonin, 2018). These properties relate to characteristics of either the picture (e.g., visual complexity) or of the associated verb. Verb characteristics thereby comprise word form properties (e.g., word length, morphological structure) and attributes pertaining to the usage of a verb by individual speakers or a language community (e.g., age of acquisition, word frequency). Moreover, normative data has to be specific for a subpopulation of speakers, as properties like age or health status affect behavioral performance (Ramsay, Nicholas, Au, Obler, & Albert, 1999; Williamson, Adair, Raymer, & Heilman, 1998).

### Picture characteristics

For the production of verbs, the number of different *answers per picture* (n_response_) and the related variables *name agreement* (NA) and *entropy of responses* (H) exert strong and robust effects on response latencies across different languages. These highly correlated response properties, summarized as *name agreement indices* (Székely et al., 2005), inversely correlate with naming latencies (Cuetos & Alija, 2003; Khwaileh et al., 2018; Schwitter et al., 2004; Shao et al., 2014; Székely et al., 2005).

In contrast, only one former action naming normative study shows that the v*isual complexity* (VC) of a picture has an independent effect on naming performance (Shao et al., 2014), whereas most other studies do not report any influence (Cuetos & Alija, 2003; Khwaileh et al., 2018; Schwitter et al., 2004; Székely et al., 2005). However, most measures of visual complexity used in previous studies are subjective and differ across studies, thus limiting comparability. Thus, several authors stress the advantage of using objective parameters, e.g., file size (Perret & Bonin, 2018; Székely & Bates, 2000).

### Verb characteristics

Besides picture attributes, several verb characteristics influence behavioral performance in action naming tasks. Many studies demonstrate that early *age of word acquisition* (AoA) predicts lower naming latencies of verbal responses, i.e., faster reaction times (Cuetos & Alija, 2003; Schwitter et al., 2004; Shao, Roelofs, & Meyer, 2014; Székely et al., 2005). In contrast, *word frequency* (FR), a measure that is strongly related to age of acquisition (Johnston & Barry, 2006), exerts inconsistent effects on naming latency, once AoA has been partialed out (Székely et al., 2005). The effect of subjectively rated *imageability* (IM) of a depicted action on behavioral performance also varies among different studies or languages (Cuetos & Alija, 2003; Khwaileh et al., 2018; Shao et al., 2014). Although frequently studied, *word length* (LE) and related measures consistently fail to predict naming latency (Cuetos & Alija, 2003; Khwaileh et al., 2018; Schwitter et al., 2004; Shao et al., 2014).

Studies in Parkinson’s disease (PD) patients suggest that behavioral performance in action naming tasks varies as a function of *motor content* (MC) of the associated verbs (Herrera & Cuetos, 2012; Herrera, Rodríguez-Ferreiro, & Cuetos, 2012). Thereby, PD patients label pictures with high motor content of the respective target word less accurately and slower than pictures with low motor content. Motor content describes “how much movement [is] needed in order to perform the actions” depicted in a stimulus (Herrera et al., 2012). Moreover, behavioral and neural data from healthy participants indicate that verbs with low and high motor content are processed differently (Grossman et al., 2002; Kemmerer, Castillo, Talavage, Patterson, & Wiley, 2008). One Turkish study provides normative data for motor content of responses to action pictures (Bayram, Aydin, Ergenc, & Akbostanci, 2017). However, up to this point, no study has systematically investigated the effect of motor content on naming latencies in healthy participants. Here, we examine the *motor content of the word* (MC_word_) and the *motor content of the picture* (MC_pic_) and their impact on naming latency.

Furthermore, the neighborhood size of a word can influence naming latency. Neighbors of a word share most of their segments, i.e., phonemes or graphemes (according to the prevalent definition all but one) (Marian, Bartolotti, Chabal, & Shook, 2012). Previous data indicate that dense phonological neighborhoods are associated with higher naming latencies (Sadat, Martin, Costa, & Alario, 2014). As grapheme to phoneme relations in German are relatively transparent, orthographic and phonological neighborhood are highly related (Marian et al., 2012). Thus, orthographic neighborhood can serve as a surrogate parameter for phonological neighborhood. A particular measure of orthographic neighborhood, the *orthographic Levenshtein distance 20* (OLD20), refers to the mean number of insertions, deletions and substitutions needed to arrive from a word at its 20 closest orthographic neighbors (Yarkoni, Balota, & Yap, 2008). In lexical decision and pronunciation tasks, low orthographic Levenshtein distance 20 correlates with lower response times (Yarkoni et al., 2008). However, previous action naming studies did not assess the effect of neighborhood size on behavioral performance. We calculate orthographic Levenshtein distance 20 for verbal responses to the action pictures. Further, we explore whether orthographic neighborhood affects response latencies in action naming.

In addition, verbs are characterized by different grammatical properties that can influence naming latencies. *Transitivity* (TR) describes the number of arguments a verb can take. Intransitive verbs do not demand an object to form a meaningful construct (e.g., “she fell”), while transitive or ditransitive verbs require one or two objects, respectively (e.g., “I need my glasses” and “He passed her the ball”). It has been found that action pictures with intransitive target names are labeled faster than those with transitive targets (Kauschke & von Frankenberg, 2008; Kauschke & Stenneken, 2008). For *reflexive* verbs, the agent is also the patient of the action (*Reflexivity* [RE], e.g., “I am washing myself”). While investigations on behavioral effects of reflexivity are missing, data from one neuroimaging study suggests that reflexive and non-reflexive verbs are processed differentially (Shetreet & Friedmann, 2012). Moreover, *morphological complexity* (CO) has been shown to influence naming latencies (Kauschke & Stenneken, 2008) as well as neuronal processing (Finocchiaro, Basso, Giovenzana, & Caramazza, 2010) for verbs. In German, a prevalent type of morphologically complexity in verbs is prefixation (Heide, Lorenz, Meinunger, & Burchert, 2010), e.g., prefix “aus” + stem “brechen” = “ausbrechen”; *to escape*). Here, we investigate possible effects of these three grammatical properties on naming latency.

In picture naming, participants may provide several different verbal responses to a given picture. Previous studies therefore restricted their analysis to the verb most frequently produced per picture, i.e., the dominant response. To this end, they excluded pictures, in which dominant responses did not account for a minimum of 40% to 50% of all answers (i.e., name agreement below 40% to 50%), leading to a lower number of items (Khwaileh et al., 2018; Shao et al., 2014). In this study, we overcome this constraint by characterizing a wide range of verbal responses that participants had provided to each picture. This enables us to conduct trial-based statistical analyses, which account for response variability among participants. Specifically, we investigate associations between naming latencies and picture as well as verb characteristics by means of repeated measures correlations and linear mixed effects modeling. Additionally, users of our database are hereby able to (1) flexibly choose the name agreement threshold that should be applied in their study and (2) incorporate verb characteristics into their analysis when the subject’s response deviated from the dominant answer.

It is important to note that the effect of picture and verb characteristics on naming performance is partially language-specific, impeding cross-linguistic application and comparison (Bates et al., 2003). A widespread literature of object naming studies has emerged for several languages (for review, see Perret & Bonin, 2018). Prior investigations of action naming in the German language were based on a substantially smaller data set and did not provide formal normative data (Kauschke & von Frankenberg, 2008). Here, we establish a normative German data set for action naming that is based on a young and healthy population. We thereby consider traditionally assessed picture characteristics, such as name agreement indices and visual complexity of the picture, as well as verb characteristics, such as age of acquisition, word frequency, imageability and word length. In addition, we report less considered variables that might impact language processing, i.e., motor content, orthographic neighborhood, transitivity, reflexivity and morphological complexity.

## Methods

Our study consisted of two experiments. In the first experiment, we conducted a timed picture naming task. We used pictures depicting actions from two freely available databases that have previously been validated for other languages (Bayram et al., 2017; Székely et al., 2005) and assessed naming latencies of the associated responses. In addition, we appraised the perceived motor content of the pictures. In a second experiment, we characterized the motor content, imageability and age of acquisition of 600 frequent German verbs including responses of the first study. Both experiments were approved by the local medical ethics committee of the Medical Faculty of the University of Marburg (study number 198/17) and were in accordance with the latest version of the Declaration of Helsinki.

### Experiment 1

#### Participants

In the first experiment, we included 59 participants (37 women, mean ± SD age: 24.6 ± 3 years, formal education: 17.7 ± 2.2 years). Participants were native speakers of German without any history of neurological or psychiatric disorders. All participants provided written informed consent.

#### Procedure

The experiment was conducted in a dimly lit soundproof testing room. We presented all stimuli on a computer screen at a fixed distance using Psychophysics Toolbox (Brainard, 1997) implemented in MATLAB 2016b (The MathWorks, Natick, MA, USA) and presented on a VG248QE monitor (Asus). Audio responses were recorded with an SF-920 microphone (Elegiant) and motor content ratings were collected via a N001 numeric keypad (Jelly Comb).

**Fig. 1:**
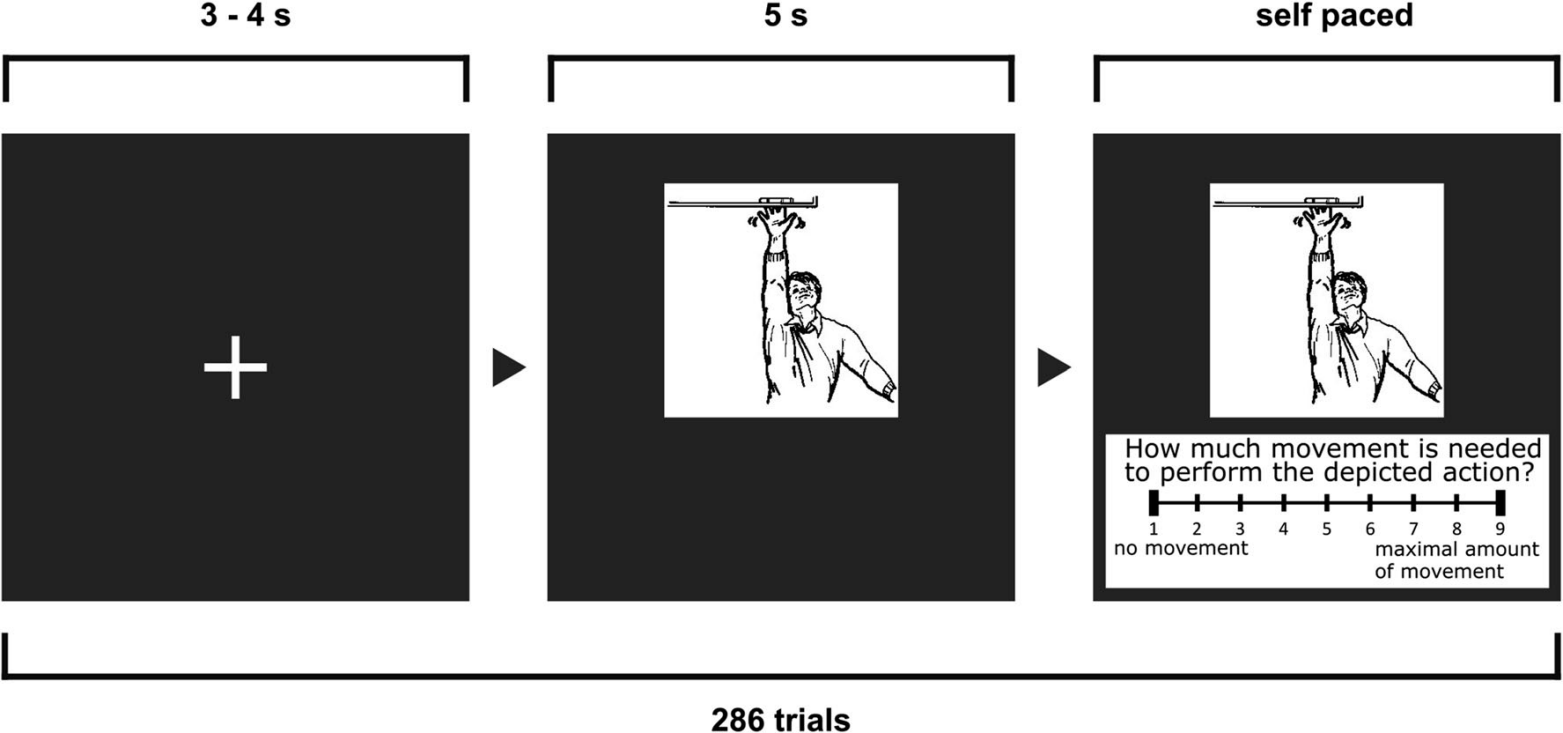
Setup of Experiment 1. Example stimulus from the IPNP database (Székely et al., 2005). English translation of the motor content rating prompt: “How much movement is needed to perform the depicted action?”

After several practice trials, which did not enter analyses, participants viewed 286 black-and-white drawings depicting actions (Fig. 1). We used a combination of action pictures from the International Picture-Naming Project (IPNP) database validated for the English language (129 pictures; Székely et al., 2005, https://crl.ucsd.edu/experiments/ipnp/) and from a recent study conducted in the Turkish language (157 pictures; Bayram et al., 2017, https://osf.io/awj3r).

The pictures were presented in randomized order in six blocks with self-paced breaks between blocks. The first five blocks comprised 50 pictures and the last block 36 pictures. We rescaled all pictures to a fixed width of 500 pixels while maintaining the original aspect ratio of each picture. Participants were instructed to label the image and vocalize their response. We asked the participants to avoid hesitations, corrections or non-lexical utterances and restrict their response to a single word. Each picture was presented for five seconds, which constituted the time limit for the participant’s response. After this period, a motor content rating scale appeared underneath the picture and participants were asked to quantify the amount of movement required for the depicted action. We used a jittered inter-stimulus-interval of 3–4 s. For both tasks, we instructed participants to respond as fast as possible.

#### Response transcription

We transcribed responses whilst taking into account the context of the respective picture. Thus, in the case of homophones, we chose the verb that most likely matched the depicted scene. Only verbs that were listed in the dictionary DUDEN (www.duden.de) as of August 2018 were considered legitimate. We further error-flagged trials with missing, non-comprehensible or late answers (> 5 s after stimulus onset) along with trials, in which a clear response onset could not be determined due to pre-response utterance, post-response self-correction or answers consisting of more than one word.

#### Outcome variables

**Naming latency** We determined naming latencies (RT) for each picture semi-manually using the standalone software CheckVocal (Protopapas, 2007).

**Name agreement indices** Next, we evaluated the responses given by the participants and quantified the distribution of responses for each picture by calculating three variables. To this end, we defined the dominant answer as the most frequent answer per picture in accordance to previous action naming studies (Shao et al., 2014; Székely et al., 2005).

First, we assessed the number of different answers across participants. Difference was defined on a purely lexical basis: Even semantically comparable words—i.e., (near) synonyms—were counted as distinct when they differed in their word-form (e.g., “einklemmen” and “klemmen”; *to jam*). Second, we calculated name agreement by dividing the number of times the most frequent response (i.e., the dominant response) was given by the total number of participants who produced an analyzable response. For example, if the dominant response “run” was given by 80% of all participants after exclusion of invalid responses, NA *=* 0.8.

1 $$NA=\frac{n\ dominant\ responses}{n\ participants}$$

Third, we assessed the distribution of responses by calculating entropy with n_response_ being the number of different answers and p_i_ being the probability of the *i*th answer occurring (Shannon & Weaver, 1998; Snodgrass & Vanderwart, 1980):

2 $$H=-\sum \limits_{i=1}^{n_{response}}{p}_i{\mathit{\log}}_2{p}_i$$

Thus, high entropy indicates a more uniform distribution of responses.

**Motor content of the picture** Participants were instructed to quantify the amount of movement required for the depicted action by button press. We assessed motor content of the picture on a nine-point scale (ratings from 1–9; 1: no movement; 9: maximal amount of movement). The rating prompt in German translates to “How much movement is needed to perform the depicted action?”

#### Additional picture characteristics

In accordance with a previous study (Székely et al., 2005), we calculated objective visual complexity based on file size (jpeg format). As stimulus dimensions differed among the two databases employed for the pictures, we first superimposed each picture onto a uniformly sized black background whose dimensions equaled the size of the largest picture.

### Experiment 2

#### Participants

In this experiment, we included 150 healthy participants (103 women, mean ± SD age: 24 ± 4.1 years, education: 16.6 ± 2.4 years). Participants were native speakers of German without any history of neurological or psychiatric disorders. All participants provided written informed consent. Participants evaluated motor content, imageability and age of acquisition of 600 German verbs using an online survey (Leiner, 2014). In order to stay within a feasible time frame per participant, we randomly split the cohort into three groups (*n* = 50 each), who rated one of the three variables of interest, respectively.

#### Procedure

Participants viewed a set of 600 verbs in a randomized order on their personal computer screen. We instructed them to rate imageability, motor content or age of acquisition (depending on group membership) of each word as fast and intuitively as possible. On each page, we presented 60 words with 10 pages in total.

As participants gave a total of 1044 unique responses in Experiment 1, we characterized a subset of the most commonly provided verbs for reasons of feasibility. For each picture from Experiment 1, we chose the minimum number of verbs that jointly represented the responses of more than half of the participants (e.g., verb A: 40% of participants, verb B: 30% of participants). For 184 pictures, a single verb accounted for at least 50% of all responses, with successively more verbs required to reach this threshold for the remaining pictures (58 pictures: two verbs, 23 pictures: three verbs, 10 pictures: four verbs, 5 pictures: five verbs, 2 pictures: six verbs and 1 picture: eleven verbs). Due to technical errors in response transcription, 3 of the 286 presented pictures had to be excluded from the data set. This resulted in a total of 299 unique words related to the pictures of Experiment 1, due to a substantial overlap of responses among the pictures. The final data set of 600 words additionally included 301 frequent German verbs from the SUBTLEX-DE database (Brysbaert et al., 2011).

#### Outcome variables

**Imageability** The imageability of a word, i.e., how easily a word is able to evoke a mental image, was assessed according to Paivio, Yuille, and Madigan (1968). We instructed participants to indicate the perceived imageability of the verb by button press using a seven-point scale (1: low imageability; 7: high imageability).

**Motor content of the word** Similarly, motor content of the word was assessed on a nine-point scale (1: lowest amount of movement; 9: highest amount of movement), analogous to Experiment 1 and previous studies (Bayram et al., 2017). Participants had to indicate motor content by button press.

**Age of acquisition** Further, we asked participants to estimate the age of acquisition of the word, i.e., the age (in years) at which they think they have understood the meaning of the word for the first time. In line with Birchenough, Davies, and Connelly (2017), we instructed participants to report a single year instead of indicating a time period.

#### Additional word characteristics

For each of the 600 words, we additionally compiled data from external sources and computed word specific properties. First, we derived word frequency from the SUBTLEX-DE database (Brysbaert et al., 2011). We exclusively used the frequency count of the lower-case word form. This was to counter overestimation of word frequency of verbs also used as nouns when capitalized (e.g., “essen” = *to eat* vs. “Essen” = *food*). Further, we calculated the orthographic Levenshtein distance 20 of our selected verbs to all words from the SUBTLEX-DE database (Brysbaert et al., 2011) and word length as number of letters. FR, OLD20 and LE were also computed for all other responses in Experiment 1 that were not assessed in Experiment 2. Additionally, two experienced linguists classified the transitivity, reflexivity and morphological complexity of each verb. All three variables were classified into binary categories, respectively (intransitive vs. transitive and ditransitive; reflexive vs. non-reflexive and partly reflexive; complex vs. non-complex).

#### Quality check

We conducted several quality checks of the data in order to assure that participants completed the survey with due diligence. First, we excluded participants who completed less than 70% of the questionnaire and who exceeded a time limit of 24 hours to complete the survey. Second, we excluded fraudulent cases, i.e., participants who exhibited unusual response patterns (high difference from median entropy and high proportion of answers outside of one median absolute deviation from the median answer) or completion time (high difference from median time to completion) by means of the Minimum Covariance Determinant (Hubert & Debruyne, 2010). These computations were performed separately for each survey.

### Statistical analysis

We hypothesized that both the attributes of the presented pictures and characteristics of the associated verbs affect the latency of verbal responses to pictures in Experiment 1. Therefore, we incorporated the variables obtained in Experiment 2, i.e., verb characteristics, into the analysis of Experiment 1. Many studies only used verb characteristics of the dominant verbal response for each picture (Cuetos & Alija, 2003; Khwaileh et al., 2018; Schwitter et al., 2004; Shao et al., 2014; Székely et al., 2005). However, particularly in cases of low name agreement, the characteristics of the dominant response only partially represent the spectrum of verbal associations for a picture. While some studies therefore introduced a cut-off for excluding stimuli with low name agreement values (Schwitter et al., 2004), we characterized a wide range of responses per picture and conducted trial-based analyses instead.

#### Data preprocessing

For all further analyses, we excluded trials with errors (4.9% of all trials), and those which contained responses given only by a single participant for one picture (7.4% of all trials). In sum, 87.7% of all trials were eligible for statistical analyses and reporting of normative data (i.e., average scores across participants), of which 91% were fully characterized in Experiment 2 (79.8% of all trials). Before calculating correlations and the linear mixed model, we transformed some variables in order to obtain normally distributed values based on visual assessment. To this end, RT, n_response_, VC, AoA, FR and OLD20 were logarithmized and IM was exponentiated. After that, multivariate outlier trials were detected using the Minimum Covariance Determinant on the basis of all trials with full available data (Hubert & Debruyne, 2010). With this procedure, we identified 2% of these trials as outliers and excluded them from further analyses. Finally, variables were z-transformed before entering them into the respective models. As 99.4% of trials fell into the pre-defined category of non-reflexive and partly reflexive verbs, we did not incorporate reflexivity in the models due to lacking variation among trials. TR and CO were coded with dummy variables (0 = intransitive / non-complex, 1 = transitive and ditransitive / complex).

#### Normative data and descriptive statistics

While correlations and the linear mixed model are based on trial-by-trial data, we additionally report averaged picture and word characteristics across participants, enabling straightforward usability as a normative data set (MC_pic_, IM, MC_word_, AoA, FR, OLD20, LE, TR, RE, and CO). For IM, MC_word_, AoA, TR, RE, and CO, we only considered trials with responses that were evaluated in Experiment 2.

We then computed descriptive statistics for each variable over all pictures.

#### Repeated measures correlations

We calculated repeated measures correlations between naming latency, picture characteristics (H, n_response_, NA, MC_pic_, VC) and verb attributes (IM, AoA, MC_word_, LE, FR, OLD20, TR, CO) using the *rmcorr* package (Bakdash & Marusich, 2017). This method takes the non-independence of repeated measures within each participant into account and estimates the common association between two variables among participants (Bakdash & Marusich, 2017). We then tested basic assumptions of linearity, homoscedasticity and normal distribution of errors using the Rainbow test, the Breusch-Pagan test, and the Kolmogorov-Smirnov test, respectively. Overall, we did not find any severe violations of these assumptions. We corrected *p* values for multiple comparisons by means of the Benjamini-Hochberg procedure (Benjamini & Hochberg, 1995) and reported weak (R ≥ 0.1), moderate (R ≥ 0.3) and strong (R ≥ 0.5) correlations following Bakdash and Marusich (2017).

#### Linear mixed effects model

Thereafter, we employed linear mixed effects modeling incorporating picture characteristics and verb attributes in order to evaluate which of these uniquely contributed to the overall variance in naming latencies explained, using the *lmer* function from the *lme4* package (Bates, Mächler, Bolker, & Walker, 2015). As fixed effects we included H, MC_pic_, VC, AoA, IM, MC_word_, FR, LE, OLD20, CO, and TR. We established a maximal random effects structure as dictated by study design (Barr, Levy, Scheepers, & Tily, 2013). We therefore incorporated by-participant random intercepts and by-participant random slopes for H, MC_pic_, VC, AoA, IM, MC_word_, FR, LE, OLD20, CO and TR. Likewise, we included by-picture random intercepts and by-picture random slopes for MC_pic_, AoA, IM, MC_word_, FR, LE, OLD20, CO and TR. Note that we did not incorporate by-picture random slopes for H and VC as these variables do not show within-picture variation. As name agreement indices are highly intercorrelated, we only included entropy of responses. The full model equation is reported in Table 1. We tested for multicollinearity of predictors by computing the variance inflation factor, which was < 2 for all variables and thus indicated that multicollinearity was not a concern. The model was iteratively estimated by the restricted maximum likelihood procedure, and *p* values were computed by *t* tests using Satterthwaite's approximation for degrees of freedom. We tested assumptions of normal distribution of residuals and homoscedasticity visually, using the *performance* package (Lüdecke, Makowski, Waggoner, & Patil, 2020). We did not detect severe violations of these assumptions.

**Table 1:** Equation of the linear mixed effects model

**Equation**	
RT~1 + H + MC_pic_ + VC + AoA + IM + MC_word_ + FR + OLD20 + LE + CO + TR + (1 + MC_pic_ + AoA + IM + MC_word_ + FR + OLD20 + LE + CO + TR | Picture) + (1 + H + MC_pic_ + VC + AoA + IM + MC_word_ + FR + OLD20 + LE + CO + TR | Participant)	

*H*  entropy, *MC*_*pic*_ motor content of the picture, *VC* visual complexity, *AoA* age of acquisition, *IM* imageability, *MC*_*word*_ motor content of the word, *FR* frequency per million, as derived from SUBTLEX-DE, *OLD20* mean orthographic Levenshtein distance of the 20 nearest neighbors, *LE* word length (in letters), *CO* morphological complexity

## Results

In Experiment 1, we excluded three participants due to technical problems during data acquisition and one participant due to naming latencies exceeding two standard deviations above the mean. Thus, we included the data of 55 participants in the statistical analysis. In Experiment 2, twenty cases were excluded due to missing data, exceedingly long time to completion or unusual response patterns (see above). Therefore, 130 data sets were confirmed eligible for statistical analysis, i.e., 44 data sets for imageability, 41 for motor content and 45 for age of acquisition.

In addition to the selected verbal responses from Experiment 1, we further included 301 frequent German verbs from the SUBTLEX-DE database (Brysbaert et al., 2011), in order to allow for a wider range of verbal responses to pictures in future experiments. Thus, we provide normative data for a total of 600 verbs (Supplementary Table 1).

### Normative data and descriptive statistics

**Fig. 2:**
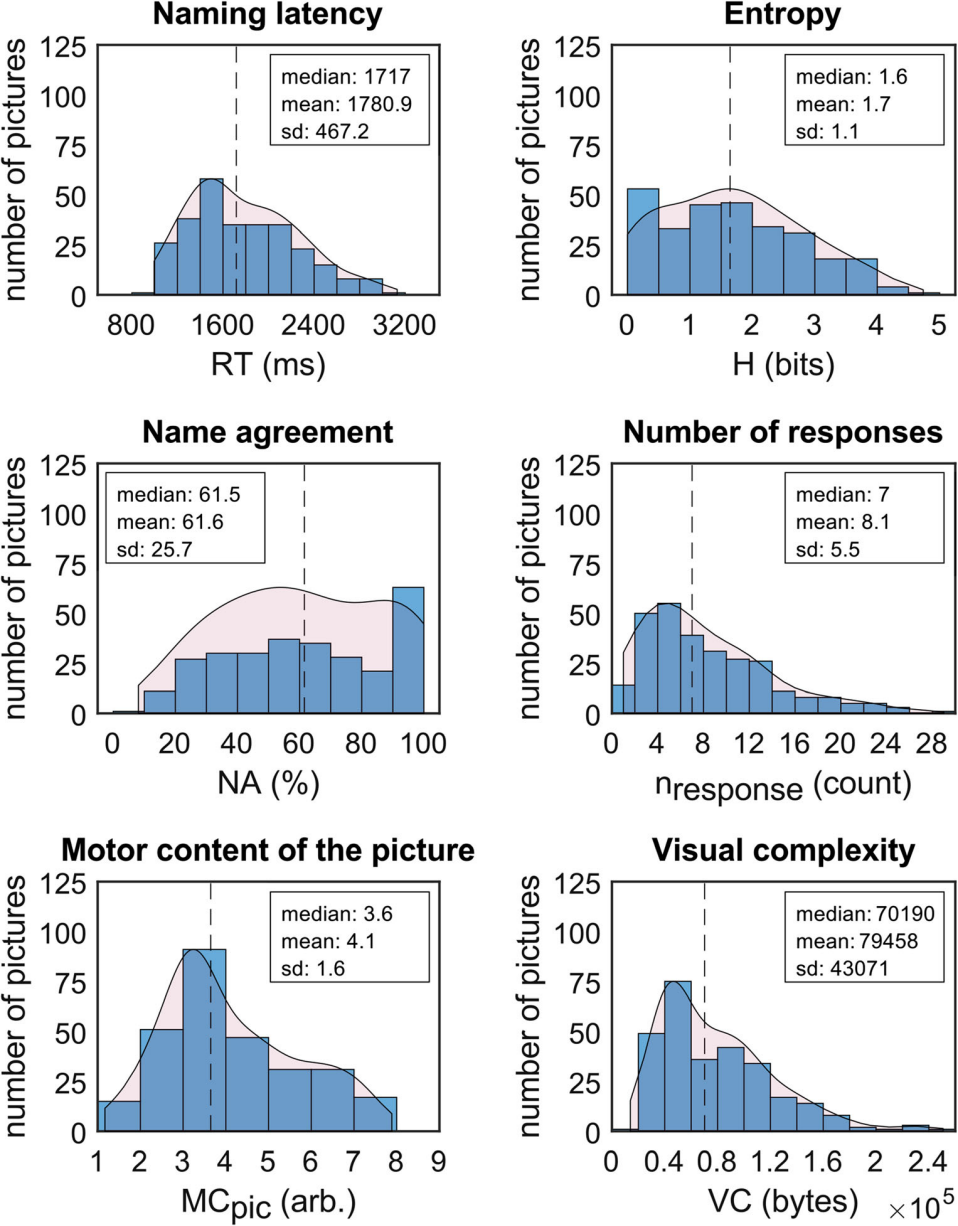
Distribution of naming latency (RT) and picture characteristics. Binned data are indicated by blue histogram bars. Fitted probability density function is depicted as red overlay. The vertical dashed line indicates the median value. RT = reaction time (i.e., naming latency), H = entropy, NA = name agreement, n_response_ = number of different answers, MC_pic_ = motor content of pictures, VC = visual complexity, arb. = arbitrary

**Fig. 3:**
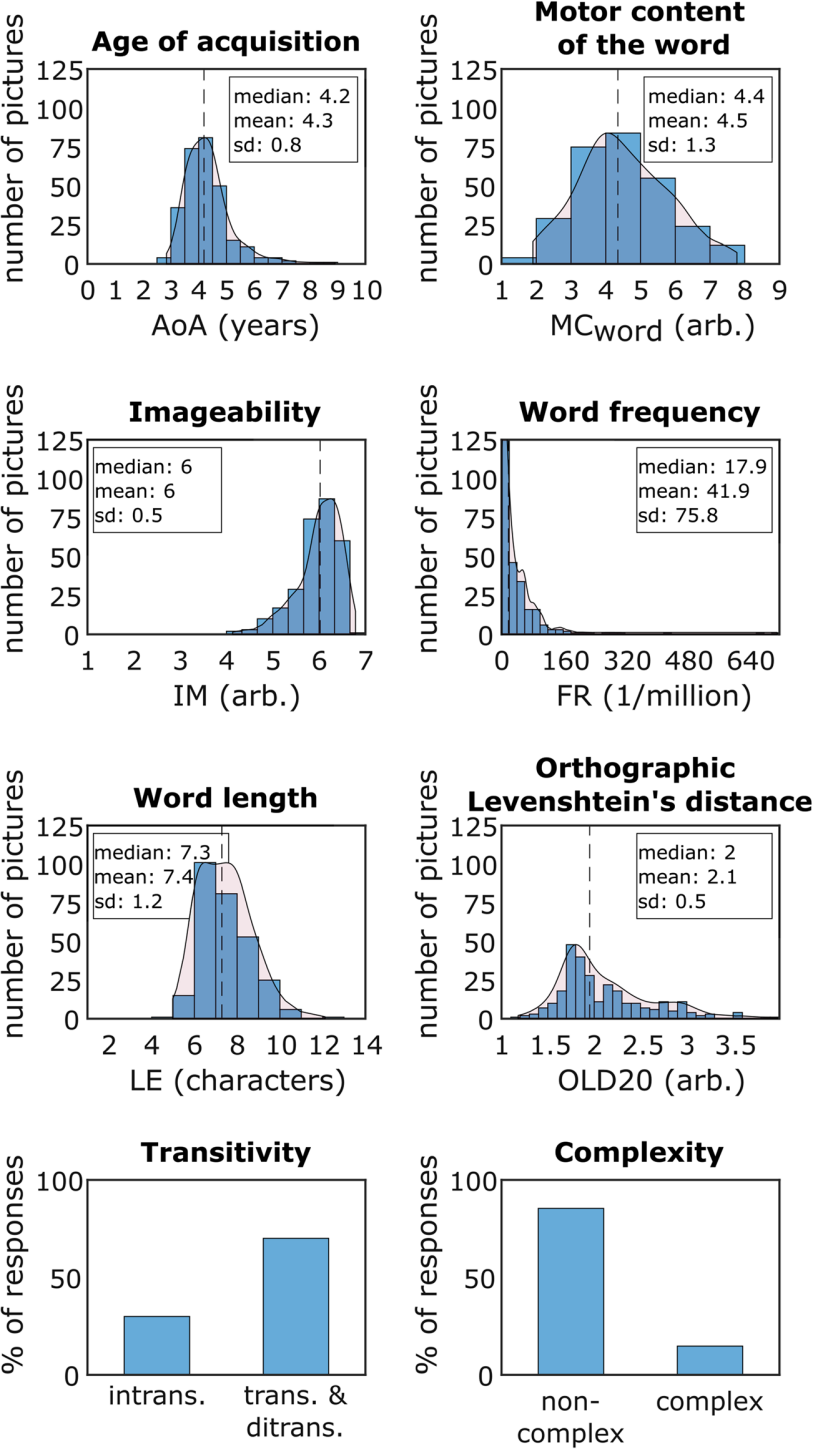
Distribution of verb characteristics. Binned data are indicated by blue histogram bars. Fitted probability density function is depicted as red overlay. The vertical dashed line indicates the median value. AoA = age of acquisition, MC_word_ = motor content of the word, IM = imageability, FR = frequency per million, as derived from SUBTLEX-DE, LE = length of answers (in letters), OLD20 = mean orthographic Levenshtein distance of the 20 nearest neighbors, arb. = arbitrary

The distributions of normative data for each variable across pictures are illustrated in Figs. 2 and 3. This normative data set is available in an online repository (see Open Practices Statement).

Descriptive statistics of the normative data are reported in Supplementary Table 2.

### Correlations of naming latency with picture and verb characteristics

**Fig. 4:**
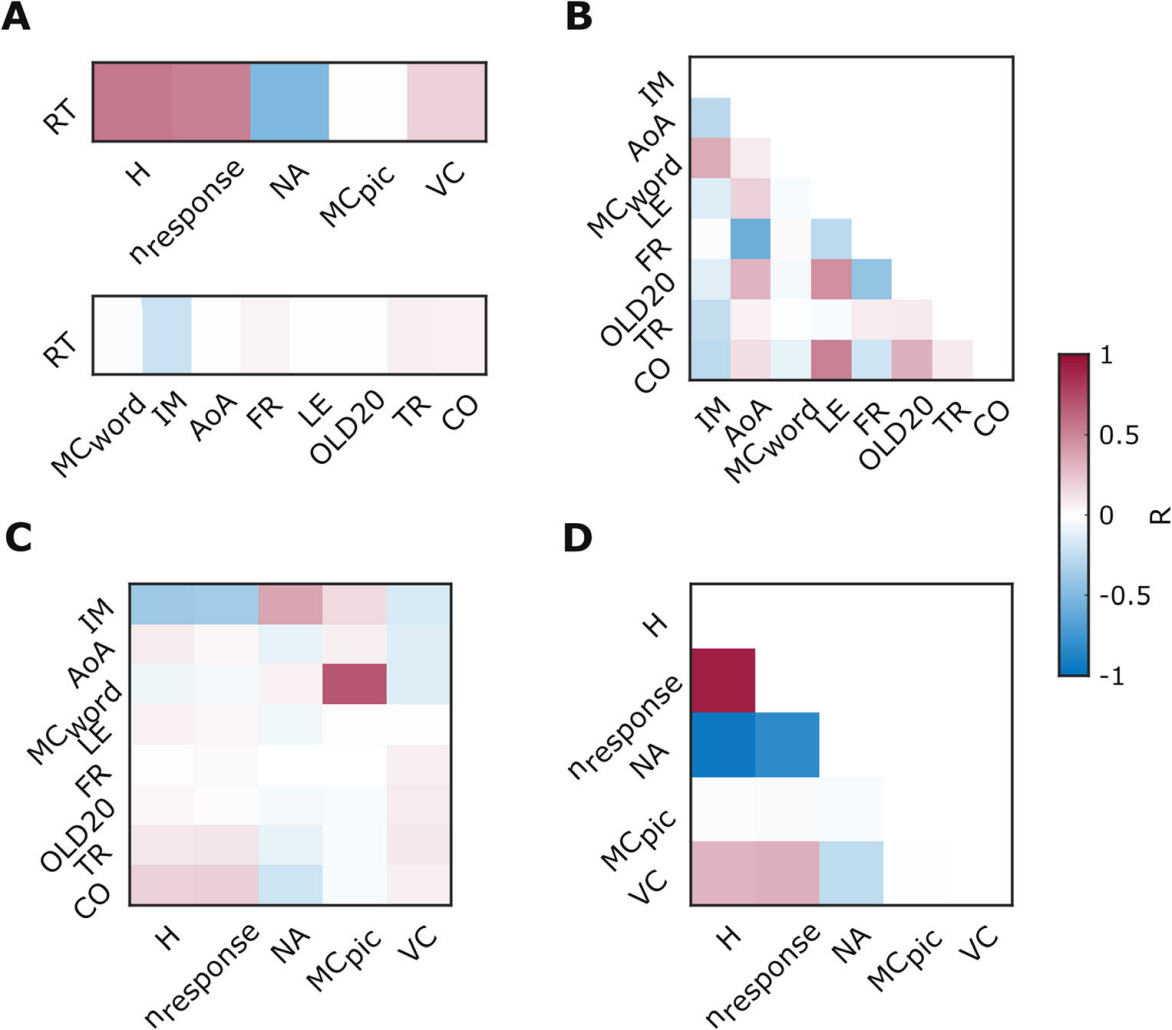
Correlational analyses between **a** naming latency and picture and verb characteristics, **b** verb characteristics, **c** picture and verb characteristics and **d** picture characteristics. RT = reaction time (i.e., naming latency), H = entropy, n_response_ = number of different answers, NA = name agreement, MC_pic_ = motor content of the picture, VC = visual complexity, MC_word_ = motor content of the word, AoA = age of acquisition, IM = imageability, LE = length of answers (in letters), FR = frequency per million, as derived from SUBTLEX-DE, OLD20 = mean orthographic Levenshtein distance of the 20 nearest neighbors, TR = transitivity, CO = morphological complexity, R = repeated measures correlation coefficient

All results from our correlation analysis are summarized in Table 2. Naming latencies strongly increased as a function of H and n_response_ and decreased with NA (Fig. 4a). Additionally, naming latencies weakly increased with higher VC and weakly decreased with increasing IM. We did not find any other correlations that at least fulfilled the criteria of a weak correlation (R ≥ 0.1).

**Table 2:** Repeated measures **c**orrelation coefficients for correlations among naming latency, picture characteristics and verb characteristics

	RT	H	n_response_	NA	MC_pic_	VC	IM	AoA	MC_word_	LE	FR	OLD20	TR	CO
RT														
H	0.55*													
n_response_	0.53*	0.92*												
NA	-0.5*	−0.95*	−0.82*											
MC_pic_	0.01	0.02	0.03*	−0.03*										
VC	0.19*	0.31*	0.33*	−0.25*	−0.01									
IM	−0.21*	−0.38*	−0.36*	0.37*	0.15*	−0.15*								
AoA	0.01	0.08*	0.04*	−0.09*	0.07*	−0.13*	−0.27*							
MC_word_	−0.02	−0.07*	−0.04*	0.06*	0.69*	−0.13*	0.35*	0.08*						
LE	−0.01	0.06*	0.04*	−0.06*	0.01	−0.01	−0.13*	0.19*	−0.03*					
FR	0.05*	0.01	0.03*	0	0	0.07*	−0.02*	−0.57*	0.03*	−0.26*				
OLD20	−0.01*	0.04*	0.02	−0.04*	−0.03*	0.08*	−0.11*	0.31*	−0.04*	0.46*	−0.42*			
TR	0.07*	0.1*	0.11*	−0.09*	−0.03*	0.1*	−0.23*	0.06*	−0.01	−0.03*	0.08*	0.09*		
CO	0.06*	0.19*	0.2*	−0.19*	−0.03*	0.07*	−0.26*	0.14*	−0.09*	0.52*	−0.18*	0.33*	0.09*	

*RT* reaction time (i.e., naming latency), *H*  entropy, *n*_*response*_ number of different answers, *NA*  name agreement, *MC*_*pic*_  motor content of the picture, *VC* Visual Complexity, *MC*_*word*_ motor content of the word, *AoA* age of acquisition, *IM* Imageability, *LE* word length (in letters), *FR* frequency per million, as derived from SUBTLEX-DE, *OLD20* mean orthographic Levenshtein distance of the 20 nearest neighbors, *TR* transitivity, *CO* morphological complexity. * = p < 0.05 after Benjamini-Hochberg correction

### Correlations between verb characteristics

We found a strong positive correlation between LE and CO and a likewise negative association between FR and AoA (Fig. 4b). OLD20 correlated positively and moderately with AoA, LE and CO. We additionally detected a moderate positive association between MC_word_ and IM and a moderate decrease of OLD20 with higher FR. Furthermore, higher AoA was accompanied by a weak increase in CO and LE, while word frequency weakly decreased with CO and LE. A small negative correlation could be observed between IM and all remaining word characteristics except FR. We did not find any other correlations that at least fulfilled the criteria of a weak correlation (R ≥ 0.1).

### Correlations between verb and picture characteristics

Our analysis revealed a strong positive association between MC_pic_ and MC_word_ (Fig. 4c). Name agreement indices mainly correlated with IM and CO: We observed moderate increases in IM for higher NA, lower H and a smaller n_response_. Similarly, we found CO to weakly decrease with NA and increase with H and n_response_. We further found positive, but only weak correlations between n_response_ and TR and between MC_pic_ and IM. Finally, VC decreased weakly with higher MC_word_, IM and AoA. We did not find any other correlations that at least fulfilled the criteria of a weak correlation (R ≥ 0.1).

### Correlations between picture characteristics

Name agreement indices all strongly correlated with each other: We observed a negative association between NA on one side and n_response_ and H on the other side, while conversely n_response_ and H correlated positively (Fig. 4d). Furthermore, VC correlated with all name agreement indices: It weakly decreased with NA, and moderately increased with H and n_response_. We did not find any other correlations that at least fulfilled the criteria of a weak correlation (R ≥ 0.1).

### Linear mixed effects model

The mixed effects model predicting naming latency revealed independent contributions of H, FR, MC_word_ and MC_pic_ (Table 3). Specifically, higher H, FR and MC_word_ predicted slower RT, whereas higher MC_pic_ predicted faster RT. All other picture or verb characteristics did not independently predict naming latency. The combination of fixed and random effects explained 58% of variance in naming latency (*R*^2^ [conditional] = 0.58), while fixed effects alone accounted for 25% (*R*^2^ [marginal] = 0.25, Table 4). Note that zero-estimates for MC_pic_ and CO (0) random effects indicate a singular fit of the model. In an additional model, we therefore excluded these terms to compute a non-singular fitted model. However, the results of this model were practically identical to the reported model (data not shown). This is in line with Brauer and Curtin (2018), who state that singular model fits do not necessarily affect fixed effects estimates.

**Table 3:** Results of the linear mixed effects model

**Fixed Effects**					
Variable	Estimate	SE	df	T	p
(Intercept)	0.01	0.07	77.1	0.1	0.92
H	0.49	0.02	240.3	20.1	< 0.001
MC_pic_	−0.04	0.01	202.5	−3.3	0.001
VC	0.02	0.02	232.6	0.9	0.35
AoA	−0.02	0.02	190.2	−0.7	0.48
IM	−0.03	0.02	237.0	−1.5	0.14
MC_word_	0.05	0.02	170.0	2.6	0.01
FR	0.07	0.02	196.4	3.1	0.002
OLD20	−0.001	0.02	154.8	−0.1	0.94
LE	−0.02	0.02	193.5	−1.2	0.22
CO (1)	−0.002	0.05	156.2	0.0	0.96
TR (1)	0.04	0.04	163.1	1.0	0.30
**Random Effects**					
Group	Variable	Variance	SD		
Picture	(Intercept)	0.016	0.128		
	MC_pic_	0	0		
	AoA	0.014	0.118		
	IM	0.012	0.111		
	MC_word_	0.006	0.079		
	FR	0.011	0.105		
	OLD20	0.009	0.095		
	LE	0.008	0.089		
	CO (0)	0.050	0.224		
	CO (1)	0.021	0.144		
	TR (0)	0.008	0.090		
	TR (1)	0.018	0.136		
Participant	(Intercept)	0.0001	0.012		
	H	0.008	0.092		
	MC_pic_	0.001	0.031		
	VC	0.0003	0.017		
	AoA	0.003	0.057		
	IM	0.001	0.024		
	MC_word_	0.0003	0.016		
	FR	0.002	0.047		
	OLD20	0.001	0.033		
	LE	0.0003	0.016		
	CO (0)	0	0		
	CO (1)	0.012	0.108		
	TR (0)	0.211	0.459		
	TR (1)	0.193	0.440		
Residual		0.439	0.663		

*H* entropy, *MC*_*pic*_  motor content of the picture, *VC* visual complexity, *AoA* age of acquisition, *IM* imageability, *MC*_*word*_ motor content of the word, *FR* frequency per million, as derived from SUBTLEX-DE, *OLD20* mean orthographic Levenshtein distance of the 20 nearest neighbors, *LE* word length (in letters), *CO* morphological complexity (0 = non-complex, 1 = complex), *TR* transitivity (0 = intransitive, 1 = transitive or ditransitive)

**Table 4:** Model fit of the linear mixed effects model

**Model fit**	
*R*^2^ (conditional)	0.58
*R*^2^ (marginal)	0.25

## Discussion

In the present study, we characterize 283 action pictures compiled from two sources (Bayram et al., 2017; Székely et al., 2005) and the associated verbal responses in German. We obtained data from 55 healthy participants and report normative values for motor content and the distribution of verbal responses to each picture. We additionally report visual complexity, measured as file size. Further, the controlled experimental setup allowed us to obtain precise naming latencies.

Moreover, we provide normative data for 600 German verbs including 299 responses from Experiment 1, as well as additional common German verbs. Three groups of 41–45 healthy participants rated imageability, motor content or age of acquisition of each verb. In addition, we report word frequency, word length, orthographic neighborhood, transitivity, reflexivity and morphological complexity for each verb.

Our data largely confirm results from previous studies regarding the relationship between picture and verb characteristics. We found a strong correlation between age of acquisition and word frequency, with more frequent words being learned earlier in life (Fig. 4b). This is a well-established effect that has already been demonstrated for German (Kauschke & von Frankenberg, 2008; Schröder, Gemballa, Ruppin, & Wartenburger, 2012) and in a range of other languages (Cuetos & Alija, 2003; Schwitter et al., 2004; Shao et al., 2014; Székely et al., 2005). We also found that name agreement increases and entropy of responses decreases moderately as a function of imageability, which are robust findings across studies investigating these variables (Cuetos & Alija, 2003; Kauschke & von Frankenberg, 2008; Khwaileh et al., 2018; Shao et al., 2014). Further, words learned at an earlier age were easier to imagine, which has also been found consistently in previous timed and untimed action naming studies (Akinina et al., 2015; Bayram et al., 2017; Cuetos & Alija, 2003; Kauschke & von Frankenberg, 2008; Khwaileh et al., 2018; Masterson & Druks, 1998; Shao et al., 2014). Together, the replication of results from German and non-German studies regarding associations among picture and verb characteristics indicate that these are robust across groups, settings and languages.

We found differential correlations of picture and verb characteristics with naming latency, such that naming latencies of visually depicted actions strongly increased with the number of different answers associated with a given picture and with its naming entropy but decreased with name agreement (Fig. 4a). These results are in accordance with previous studies investigating action naming. Several studies report similar correlations between naming latency and name agreement indices (Cuetos & Alija, 2003; Kauschke & von Frankenberg, 2008; Khwaileh et al., 2018; Schwitter et al., 2004; Shao et al., 2014; Székely et al., 2005), as well as imageability (Kauschke & von Frankenberg, 2008; Khwaileh et al., 2018; Shao et al., 2014). Our linear mixed model analysis confirmed the effect of entropy of responses on naming latency. This goes in line with a range of picture naming studies for actions and objects in other languages (Perret & Bonin, 2018).

In addition, linear mixed modeling revealed that naming latencies were longer for more frequent words. Most previous normative action naming studies did not find an independent contribution of word frequency to naming latency (Cuetos & Alija, 2003; Kauschke & von Frankenberg, 2008; Khwaileh et al., 2018; Schwitter et al., 2004). In these experiments, the authors used restrictive measures of word frequency based on news articles or books. However, recent studies showed that word frequency derived from television and film subtitles, as used in this study, is superior to estimates based on written sources (Brysbaert et al., 2011; Brysbaert & New, 2009). The higher precision of word frequency estimates may have allowed us to reveal the predictive value of word frequency for naming latency. The somewhat unexpected relationship between higher word frequency and slower naming responses is in line with one previous action naming study (Székely et al., 2005). These authors speculated that participants fall back on high-frequency multipurpose verbs for difficult items, leading to inflated reaction times due to the more effortful (and ultimately unsuccessful) search for a specific verb (Székely et al., 2005). However, this hypothesis remains to be tested in future studies, for example by obtaining parameters capturing semantic specificity. In addition, the relatively weak effect of word frequency in our study warrants further replication in other languages with contemporary word frequency measures and should be interpreted with caution.

In contrast to previous normative action naming studies (Cuetos & Alija, 2003; Schwitter et al., 2004; Shao et al., 2014; Székely et al., 2005), we did not find an effect of age of acquisition on naming latency. A large body of evidence shows that age of acquisition correlates with word frequency (Johnston & Barry, 2006). If one follows the assumption that frequent words occasionally served as fallback verbs for more difficult pictures, one could speculate that these items, which are associated with slow reaction times, obscured the otherwise expected positive correlation between AoA and naming latencies. Furthermore, imageability did not exert an independent effect on naming latency, as previously shown (Kauschke & von Frankenberg, 2008). This suggests that the effect of imageability on naming latency is mediated by name agreement indices.

Beyond the standard variables reported in action naming studies in other languages, we report perceived motor content of the action pictures (Experiment 1) and their associated verbal responses (Experiment 2). Both variables were highly inter-correlated and positively correlated with imageability. An unexpected finding was the opposing effects of MC_pic_ and MC_word_ on RT in the linear mixed model. While high MC_pic_ was associated with faster naming latencies, the contrary was the case for MC_word_. However, both parameter estimates were small in relation to the effect of H. To our knowledge, both MC_pic_ and MC_word_ have not yet been formally studied in action naming normative studies. A possible dissociation between MC_pic_ and MC_word_ may be investigated in future studies. Moreover, motor content is an interesting variable for studies in patients with movement disorders. Herrera and Cuetos (2012) showed that PD patients without dopaminergic medication exhibited slower responses to pictures with high compared to low motor content . This suggests overlapping neural networks for the processing of motor language and movement (Bak, 2013).

A second rarely reported variable, the orthographic Levenshtein distance 20, positively correlated with word length and age of acquisition and negatively with word frequency, corroborating previous findings (Yarkoni et al., 2008). However, we did not find an effect of orthographic Levenshtein distance 20 on naming latencies, in contrast to the findings of Yarkoni et al. (2008). Morphological complexity and transitivity both showed no considerable association with naming latency. As to be expected, complex verbs were longer and exhibited a sparser orthographic neighborhood than non-complex words, but they were also less imaginable.

To facilitate reproduction of results or reanalyses of our data, we provide data for all trials and subjects in an online repository (see Open Practices Statement). Further, we provide normative data for each picture by means of averaged values across participants in order to facilitate future utilization of the data set. Additionally, all responses with corresponding response frequencies are reported alongside verb characteristics for the full 600 verbs assessed in Experiment 2.

In summary, we provide the first data set of picture and verb characteristics for a compilation of 283 freely available action pictures (Bayram et al., 2017; Székely et al., 2005) for the German language. Additionally, we characterize a set of 600 action verbs. We found very similar relationships between picture and verb characteristics in comparison to action naming studies in other languages, indicating high construct validity. Entropy of responses, motor content of pictures and words, and word frequency constituted independent predictors of naming latency. Thus, future timed picture naming studies should consider controlling for these variables when assessing behavioral performance. We believe that this normative data set including standard and new parameters will be useful for future behavioral and neuroscientific studies on the cognitive processes underlying action naming.

## Supplementary Information

ESM 1 (DOCX 27.8 kb)
