# Supplementary material for: German normative data with naming latencies for 283 action pictures and 600 action verbs
Source: Behav Res Methods. 2021 Aug 2;54(2):649–62. doi: 10.3758/s13428-021-01647-w (PMC9046323; doi:10.3758/s13428-021-01647-w)
Supplement: Supplementary file 1 — (DOCX 27.8 kb) [file 13428_2021_1647_MOESM1_ESM.docx]

**Supplementary Material to:**

Busch et al.: German normative data with naming latencies for 283 action pictures and 600 action verbs

| **Variable** | **Mean** | **Standard**  **deviation** | **Min** | **Max** |
| --- | --- | --- | --- | --- |
| MC_word_ (1–9) | 3.8 | 1.3 | 1.4 | 7.8 |
| AoA (years) | 4.9 | 1.3 | 2.8 | 10.4 |
| IM (1–7) | 5 | 1.2 | 1.7 | 6.7 |
| LE | 7.3 | 1.8 | 3 | 14 |
| FR | 67.7 | 271 | 0.04 | 4201.4 |
| OLD20 | 1.9 | 0.5 | 1 | 4.7 |
|  | | **Frequency** | | |
| TR | |  | | |
| - Intransitive | | 26.5 % | | |
| - Transitive and ditransitive | | 73.5 % | | |
| RE | |  | | |
| - Non-reflexive and partly reflexive | | 99.3 % | | |
| - Reflexive | | 0.7 % | | |
| CO | |  | | |
| - Non-complex | | 84.7 % | | |
| - Complex | | 15.3 % | | |

**Supplementary Table 1** Descriptive statistics of all 600 verbs assessed in Experiment 2. MC_word_ = motor content of the word, AoA = age of acquisition, IM = imageability, LE = word length (in letters), FR = frequency per million, as derived from SUBTLEX-DE, OLD20 = mean orthographic Levenshtein distance of the 20 nearest neighbors, TR = transitivity, RE = reflexivity, CO = morphological complexity

| **Variable** | **Mean** | **Standard deviation** | **Min** | **Max** |
| --- | --- | --- | --- | --- |
| RT (ms) | 1780.9 | 467.2 | 995.6 | 3129.4 |
| H | 1.7 | 1.1 | 0 | 4.7 |
| n_response_ | 8.1 | 5.5 | 1 | 29 |
| NA (%) | 61.6 | 25.7 | 8.1 | 100 |
| MC_pic_ (1–9) | 4.1 | 1.6 | 1.2 | 7.9 |
| VC (bytes) | 79458.9 | 43071.8 | 13948 | 250804 |
| MC_word_ (1–9) | 4.5 | 1.3 | 1.9 | 7.7 |
| AoA (years) | 4.3 | 0.8 | 2.8 | 8.8 |
| IM (1–7) | 6 | 0.5 | 4.1 | 6.7 |
| LE | 7.4 | 1.2 | 4.9 | 12 |
| FR | 41.9 | 75.8 | 0.2 | 705.7 |
| OLD20 | 2.1 | 0.5 | 1.2 | 4 |
|  | | **Frequency** | | |
| TR | |  | | |
| - Intransitive | | 29.9% | | |
| - Transitive and ditransitive | | 70.1% | | |
| RE | |  | | |
| - Non-reflexive and partly reflexive | | 99.3% | | |
| - Reflexive | | 0.7% | | |
| CO | |  | | |
| - Non-complex | | 85.4% | | |
| - Complex | | 14.6% | | |

**Supplementary Table 2** Descriptive statistics of naming latency and picture and verb characteristics for 283 action pictures. RT = reaction time (i.e., naming latency), H = entropy, n_response_ = number of different answers, NA = name agreement, MC_pic_ = motor content of the picture, VC = visual complexity, MC_word_ = motor content of the word, AoA = age of acquisition, IM = imageability, LE = word length (in letters), FR = frequency per million, as derived from SUBTLEX-DE, OLD20 = mean orthographic Levenshtein distance of the 20 nearest neighbors, TR = transitivity, RE = reflexivity, CO = morphological complexity
